# Total Metabolic Tumor Volume Is a Strong Independent Prognostic Factor in Follicular Lymphomas: Results From a Sub‐Study of the FOLL12 Trial

**DOI:** 10.1002/ajh.27711

**Published:** 2025-05-14

**Authors:** Rexhep Durmo, Stephane Chauvie, Carla Minoia, Fabrizio Bergesio, Federico Fallanca, Simona Peano, Luigi Marcheselli, Antonella Anastasia, Carola Boccomini, Paolo Corradini, Jacopo Olivieri, Luca Arcaini, Federica Cavallo, Adalberto Ibatici, Luca Nassi, Vittoria Tarantino, Antonello Pinto, Caterina Stelitano, Alessandro Pulsoni, Francesca Ricci, Salvatrice Mancuso, Emanuele Cencini, Nicola Di Renzo, Clara Mannarella, Angelo Palmas, Pierluigi Zinzani, Caterina Bocci, Francesca Rossi, Angelo Michele Carella, Massimo Federico, Annibale Versari, Luca Guerra, Stefano Luminari

**Affiliations:** ^1^ Nuclear Medicine Azienda USL IRCCS of Reggio Emilia Reggio Emilia Italy; ^2^ Santa Croce e Carle Hospital Medical Physics Cuneo Italy; ^3^ Hematology Unit IRCCS Istituto Tumori ‘Giovanni Paolo II’ Bari Bari Italy; ^4^ IRCCS San Raffaele Scientific Institute Nuclear Medicine Milan Italy; ^5^ Nuclear Medicine Division S. Croce e Carle Hospital Cuneo Italy; ^6^ Fondazione Italiana Linfomi Modena Italy; ^7^ Department of Hematology ASST Spedali Civili Brescia Italy; ^8^ SC Ematologia, Dipartimento di Ematologia Ed Oncologia AOU Città Della Salute e Della Scienza di Torino Torino Italy; ^9^ Ematologia Istituto Nazionale Tumori IRCCS Milano Italy; ^10^ Ematologia, Az Osp di Udine Udine Italy; ^11^ Department of Molecular Medicine, University of Pavia & Division of Hematology Fondazione IRCCS Policlinico san Matteo Pavia Italy; ^12^ Dipartimento di Biotecnologie Molecolari e Scienze per la Salute Università di Torino Torino Italy; ^13^ Ematologia e Terapie Cellulari IRCCS Ospedale Policlinico San Martino Genova Italy; ^14^ Ematologia, Az Ospedaliera Careggi Firenze Italy; ^15^ Ematologia, Ospedale Villa Sofia Palermo Italy; ^16^ Hematology‐Oncology and Stem Cell Transplantation Unit, Istituto Nazionale Tumori IRCCS‐Fondazione ‘G. Pascale’ Naples Italy; ^17^ Hematology Grande Ospedale Metropolitano Bianchi‐Melacrino‐Morelli Reggio Calabria Italy; ^18^ Hematology, Università Sapienza Roma Italy; ^19^ Ematologia Humanitas Cancer Center Rozzano Italy; ^20^ Department PROMISE University of Palermo Palermo Italy; ^21^ UOC Ematologia Azienda Ospedaliera Universitaria Senese & University of Siena Siena Italy; ^22^ Ematologia, Azienda Ospedaliera Vito Fazzi Lecce Italy; ^23^ Ematologia Matera Italy; ^24^ Ematologia e Trapianto di Midollo Osseo Ospedale ‘San Francesco’ Nuoro Matera Italy; ^25^ IRCCS Azienda Ospedaliero‐Universitaria di Bologna Istituto di Ematologia “Seràgnoli” Seràgnoli Italy; ^26^ Dipartimento di Scienze Mediche e Chirurgiche Università di Bologna Bologna Italy; ^27^ U.O.S.D Ematologia Con Autotrapianto Cellule Staminali Ospedale di Civitanova Marche Civitanova Marche Italy; ^28^ Hematology Policlinico di Milano Milano Italy; ^29^ Ematologia Casa Sollievo Della Sofferenza Foggia Italy; ^30^ Department Chimomo Università di Modena and Reggio Emilia Modena Italy; ^31^ Nuclear Medicine Unit Fondazione IRCCS San Gerardo Dei Tintori Monza Italy; ^32^ University of Milano Bicocca Milan Italy; ^33^ Hematology Azienda USL IRCCS of Reggio Emilia Reggio Emilia Italy

**Keywords:** FOLL12 trial, follicular lymphoma, PET/CT, prognosis, total metabolic tumor volume

## Abstract

Discordant results have been generated regarding the prognostic role of Total Metabolic Tumor Volume (TMTV) in Follicular Lymphoma (FL). The use of prospective data and the adoption of the newly defined standardized SUV4 method for calculating TMTV may generate stronger evidence. We conducted a pre‐planned post hoc analysis of the prospective multicenter randomized phase III FOLL12 trial for newly diagnosed high tumor burden FL (grade 1–3a), which mandated baseline staging with PET. Baseline PET/CT scans were reviewed centrally, and TMTV was calculated using the fixed threshold of SUV4. Kaplan–Meier and Cox regression were used for survival analysis. The primary study endpoint was Progression free Survival (PFS). A total of 689 FL patients were available for TMTV definition. Median TMTV was 161 mL (IQR 50 to 388 mL) and the best cutoff value was set at 180 mL. Patients with high TMTV had a significantly lower 5‐year PFS compared to those with low TMTV: 59% (95% CI, 53–65%) vs. 74% (95% CI, 69–78%) HR 1.61 (95% CI, 1.24–2.09). Prognostic role of TMTV was independent of study arm, chemotherapy regimen, and FLIPI2. Combined with FLIPI‐2, we identified three groups with different 5‐yr PFS rates, with the lowest rates (51%) for patients with high TMTV and high FLIPI2. Combined TMTV and FLIPI model was also prognostic to predict the risk of early progression and of death. Applying the SUV4 standard method pre‐treatment TMTV is confirmed as a strong and independent predictor of PFS in FL patients. Integrating TMTV with FLIPI‐2 improves risk assessment.

## Graphical abstract

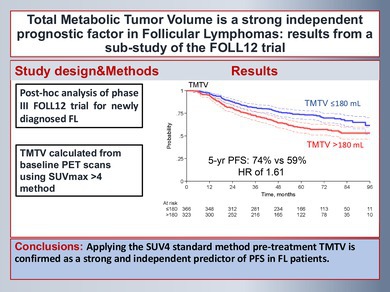

## 1 Introduction

Follicular Lymphoma (FL) is the second most common type of non‐Hodgkin lymphoma (NHL), characterized by slow‐growing malignant B‐cell proliferation [1]. Despite the high response rates to current therapies and excellent observed outcomes, FL presents a heterogeneous clinical course and the early and accurate identification of high‐risk patients is still identified as an unsolved problem [2, 3, 4, 5].

In the past few years, the use of 18F‐fluorodeoxyglucose positron emission tomography/computed tomography (FDG PET/CT) has gained widespread acceptance for the evaluation of FL either as a staging or restaging procedure at the end of treatment [6, 7, 8, 9]. Among PET‐derived measures, baseline total metabolic tumor volume (TMTV) has emerged as a promising prognostic biomarker in various lymphoma subtypes including FL [10]. In a first retrospective study conducted on a retrospective series of 185 patients, high TMTV defined using a cut‐off of 510 mL was associated with shorter survival compared to low TMTV patients [11]. A subsequent retrospective analysis conducted on FL enrolled in the GALLIUM study (presented as an abstract) was not able to confirm the prognostic role of TMTV, reducing the enthusiasm about the real usefulness of TMTV as a prognosticator in FL [12]. In a recent retrospective analysis of a subset population obtained from the Relevance, the prognostic role for TMTV was finally reaffirmed in patients treated with immunochemotherapyc [13]. Available discordant evidence has been generated by retrospective analyses and using nonstandard segmentation protocols to define TMTV, aiming for additional data on additional series of patients [14, 15]. In this context, we aimed to investigate the prognostic role of TMTV as a subset analysis of the FOLL12 trial, which is a multicenter, randomized, phase III trial that compared standard vs. response‐adapted maintenance therapy in patients with grade 1–3a advanced stage FL [16]. The study included a prospective and preplanned centralization of baseline FDG PET/CT for all enrolled patients, allowing for the assessment of TMTV on patients' outcomes. In addition to the prospective design of this study, we provide for the first time TMTV data defined using the SUVmax ≥ 4 contouring method recently approved as international standard [17].

## 2 Material and Methods

### 2.1 Study Population

The present study was conducted as a post hoc analysis of the prospective multicenter randomized phase III FOLL12 trial [16]. Briefly, the trial included previously untreated patients aged 18–75 years who had a confirmed diagnosis of FL grade 1, 2, or 3a according to the 4th edition of WHO classification [1] had Ann Arbor stage II‐IV, an Eastern Cooperative Oncology Group (ECOG) performance status of 0–2, and a FLIPI2 greater than 0. All patients had to have High tumor burden FL according to GELF criteria [18]. Patients were randomized before the start of induction therapy to receive a standard or experimental treatment. In both study arms, induction therapy consisted of six cycles of either Rituximab in combination with cyclophosphamide, doxorubicin, vincristine, and prednisone (R‐CHOP) or bendamustine (R‐B), as determined by the local investigator. After completion of induction therapy, response to treatment was evaluated with FDG PET/CT and molecular assessment of t(14;18) chromosomal translocation by polymerase chain reaction (PCR) on marrow and peripheral blood. For metabolic response, Lugano criteria with Deauville score (DS) were adopted and defined upon centralized review of PET scans [19]. All patients randomized to the standard arm had to receive 12 doses of rituximab maintenance regardless of their metabolic response. Post induction therapy in the experimental arm was observation for patients achieving complete metabolic response (DS1‐3), weekly rituximab up to 12 doses for patients with molecular residual (MRD+) or relapse, and radioimmunotherapy followed by rituximab maintenance for patients not achieving a metabolic response (DS4‐5) [16].

The evaluation of the prognostic role of baseline PET was included among predefined secondary objectives, and centralization of baseline PET scans was required by the protocol. For the purposes of the current analysis, in addition to the FOLL12 inclusion and exclusion criteria, patients were required to have a baseline FDG PET/CT scan eligible for central review and TMTV definition.

The main study endpoint was defined as Progression Free Survival; Overall Survival and Response to therapy were identified as secondary study endpoints. Response to therapy was calculated according to international criteria [19].

### 2.2 TMTV Calculation

To be included in this study, baseline PET scans were acquired according to current EANM guidelines for PET imaging of tumors on a PET/CT scanner that were accredited through FIL clinical trial qualification [20, 21]. The WIDEN platform was utilized to centrally collect baseline FDG PET/CT image data in anonymized Digital Imaging and Communications in Medicine (DICOM) format, uploaded by the local center in real time [22]. Then, PET images were used for the prospective calculation of TMTV. Analysis of TMTV was performed by three expert nuclear medicine physicians (AV, LG, and FF), who analyzed a randomized one third of the population each. The assessment of the reproducibility of TMTV measurements among the readers was conducted on a subset of patients randomly selected, comprising 20% of the total study population. TMTV was computed using the free semiautomatic software Beth Israel Fiji (http://petctviewer.org). TMTV was obtained from baseline scans by summing the metabolic volumes of all individual nodal and extra nodal lesions that were segmented using a semiautomated method, applying a fixed SUV of 4 (SUVmax ≥ 4) [17]. Volumes including physiological uptake areas or uptake areas not correlated to lymphoma were excluded from TMTV calculation by the operator. Bone marrow involvement was included in volume measurement only if there was focal uptake. The spleen was considered as involved if there was focal uptake or diffuse uptake higher than 150% of the liver background [23].

### 2.3 Statistical Analysis

Continuous variables are reported as median, range, and interquartile range (IQR); categorical variables are reported as absolute and percentage frequencies for the total number of cases. The proportion is reported according to the exact binomial distribution with the 95% confidence interval (95% CI). The distribution of time‐to‐event functions was estimated by using the Kaplan–Meier product‐limit method. Comparisons of categorical variables between two or more groups were performed using Fisher's exact test or Chi‐squared test, when appropriate. The comparison of continuous covariates between any two groups was performed by means of the Mann–Whitney test. The comparison of survival functions between two or more groups was performed using the log‐rank test, and the effect of covariates was expressed as hazard ratio (HR) estimated by Cox proportional hazard (PH) regression model with its 95% CI.

Three different approaches were used to define the cutoff for survival prediction for TMTV, stratified by random arm: maximum log‐rank statistic [24], area under curve receiver operating characteristic (AUC‐ROC) analysis for survival data [25] according to Liu method, and Cox PH regression restricted cubic spline (RCS) used to evaluate the continuous nonlinear relationship between TMTV and PFS risk.

The knots in TMTV for the restricted cubic spline function were selected by minimum AIC (Akaike index criterion) after 1000 bootstrap resamples of Cox PH regression adjusted by FLIPI‐2, sex, induction treatment, and stratified by arm, with knots ranging from 3 to 7 and placed at quantile positions as recommended by Harrel [26]. The cut‐off was internally validated by means of a bootstrap procedure: from the bootstrap samples, the stability of the cut‐off selected by RCS was evaluated, while in the out‐ofof‐bootstrap sample (OOB, test set that exclude about 37% of record from original data) the possible repeatability of the cut‐off was expressed as HR with 95% CI.

The c‐Harrell index was employed to evaluate the improvement in discrimination for PFS by TMTV compared with additional prognostic factors, stratified by random arm, and adjusted by sex and induction treatment. The reproducibility of TMTV was evaluated among the readers. The inter‐reader agreement was determined by the Bland–Altman test.

All reported *p* values are two‐sided.

#### 2.3.1 Ethical Considerations

This study was conducted in accordance with the Declaration of Helsinki and Good Clinical Practice guidelines. The FOLL12 trial (NCT02063685) was approved by the institutional review boards at each participating center, and all patients provided written informed consent before enrollment.

#### 2.3.2 Data Sharing Statement

Individual participant data that underlie the results reported in this article, after de‐identification, can be made available to investigators for research purposes on a case‐by‐case basis following the time of this publication and in accordance with the FIL Data Sharing Policy.

## 3 Results

### 3.1 Study Population Characteristics

Between December 2012 and March 2018, the FOLL12 trial enrolled 786 patients, and 689 of them (85% of the whole population) with a baseline PET scan available for central review were included in the current analysis. Ninety‐seven patients were excluded, primarily due to discrepancies found during the quality control assessment of PET/CT DICOM files, including incomplete scans or missing fields, or because the scan was not properly uploaded in the WIDEN platform for central revision. The baseline characteristics of the study population showed no significant differences comparing patients included with those not included in the analysis (Table S1 of patient's characteristics with and without PET; see Supporting Information).

Patients' characteristics are described in Table S2. After induction therapy, 553 patients achieved complete metabolic response (CMR, 80%, 95% CI, 77 to 83%) with an overall response rate (ORR) of 94% (95% CI, 92 to 96%). FLIPI2 was high (3‐ risk factors) in 35% of the patients.

Overall, the median TMTV was 161 mL (IQR 50 to 388 mL). Higher median TMTV values were associated with adverse clinical features, including anemia, high B2M or LDH or LoDLIN, greater than 6 cm, and with high risk according to FLIPI and FLIPI2 (Table S2). Median TMTV showed no significant difference between the randomized arms (*p* = 0.349) and induction treatments (Table S2). The reproducibility of TMTV calculation between different readers was found to be high being the coefficient variation in the Bland–Altman test < 10%.

### 3.2 Progression Free Survival and TMTV

With a median follow‐up of 57 months (95% CI 55–58; range 1–97 months), we observed 231/689 disease progressions or deaths, resulting in a 5‐year PFS of 67% (95% CI, 63 to 70%).

We first evaluated TMTV as a linear continuous variable, which was associated with the risk of PFS, showing a HR of 1.25 (95% CI 1.11 to 1.41, *p* < 0.001) by any increase of 500 mL when the analysis was stratified by randomized arm. We then applied three different methods to define the best prognostic cut‐off for TMTV; using maximally log‐rank test and RCS Cox regression for PFS we obtained the two different cut‐off values: 235 mL and 181 mL, respectively. While using the AUC‐ROC the cut‐off was 171 mL. Finally, the cut‐off > 180 mL was then chosen because it was associated with the best performances when applied to patients treated with standard therapy (i.e., induction Immunochemotherapy followed by maintenance) (see Supporting Information for the procedures about the evaluation of the thresholds; Figures S1, S2, S3 and Tables S3 and S4). Only 9% of patients had values between 180 and 240 mL and this small group had PFS similar to the high‐risk (32 patients) in the standard arm but its PFS was similar to the low‐risk group in the experimental arm (29 patients) Figure S4. Therefore, it was grouped with the high risk as the standard arm was the winning arm in FOLL‐12. The original cut‐off of 510 mL, obtained with the 41% SUVmax threshold method, suggested by available retrospective studies was also tested showing unstable results (See Figure S5).

**FIGURE 1:**
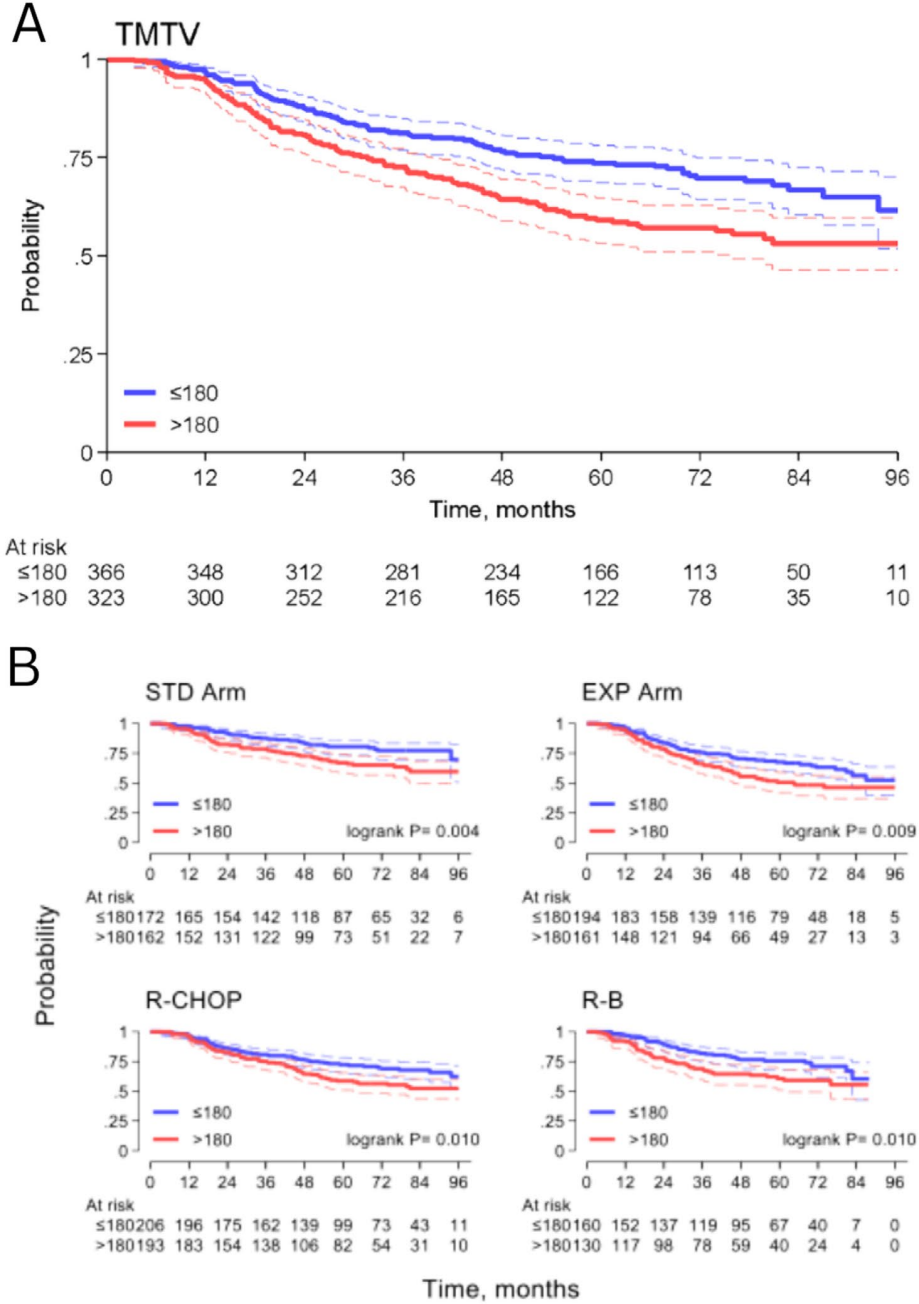
Progression‐free survival probability for patients with high (> 180 ml) and low (< 180 ml) TMTV (A). Progression free survival by TMTV level stratified by randomization arm (B, C) and induction treatment (E, D). [Color figure can be viewed at wileyonlinelibrary.com]

A univariate analysis was then conducted using the defined cut‐off for TMTV and including also other clinical and laboratory variables (Table 1). Five‐year PFS rate was significantly lower for patients with high TMTV compared with those with low TMTV: 59% (95% CI, 53 to 65%) and 74% (95% CI, 69 to 78%), respectively, with HR = 1.61 (95% CI, 1.24 to 2.09, *p* < 0.00, Figure 1A).

**TABLE 1:** Univariable and multivariable Cox PH regression of progression‐free survival (*n* = 692, fail 231).

	*n* (%)	Univariable		Multivariable			
Covariate		HR (95% CI)	*p*	HR (95% CI)	*p*	HR (95% CI)	*p*
TMTV > 180 mL	323 (47)	1.61 (1.24–2.09)	< 0.001	1.38 (1.05–1.81)	0.020	1.37 (1.03–1.81)	0.028
Age > 60	341 (50)	1.38 (1.06–1.79)	0.015			1.35 (1.03–1.76)	0.028
Sex male	317 (46)	1.23 (0.95–1.59)	0.119	1.32 (1.02–1.72)	0.036	1.39 (1.07–1.82)	0.015
B2M > ULN	378 (55)	1.57 (1.20–2.05)	0.001			1.26 (0.94–1.68)	0.124
BM+	381 (55)	1.57 (1.20–2.06)	0.001			1.49 (1.12–1.98)	0.007
LoDLIN > 6 cm	382 (55)	1.29 (0.99–1.68)	0.060			1.38 (1.04–1.83)	0.026
Hb < 12 mg/dL	111 (16)	1.74 (1.28–2.37)	0.001			1.77 (1.27–2.46)	0.001
FLIPI‐2 High	278 (40)	2.25 (1.73–2.91)	< 0.001	2.11 (1.61–2.77)	< 0.001		
Experimental arm	355 (52)	1.70 (1.30–2.21)	< 0.001				
R‐B	290 (42)	0.97 (0.74–1.26)	0.821	1.02 (0.78–1.34)	0.869	1.04 (0.79–1.37)	0.765

*Note*: In multivariable analysis the Cox PH regression was stratified by randomization arm; B2M, beta2 microglobuline; BM, bone marrow involvement; FLIPI, follicular lymphoma international prognostic index; Hb, hemoglobin; LoDLIN, longest diameter of the largest involved node; R‐B, rituximab plus bendamustine; ULN, upper limit of normality.

The prognostic role of TMTV was confirmed in both the standard and the experimental arm of the FOLL12 study (Figure 1B,C); HR of 1.84 (95% CI, 1.20 to 2.81, *p* = 0.005) and of 1.55 (95% CI, 1.11 to 2.15, *p* = 0.010), respectively, without an effect modifier (interaction term *p* = 0.526).

Moreover, interaction with induction treatment resulted in a HR of 1.53 (95% CI, 1.10 to 2.14, *p* = 0.012) in R‐CHOP and 1.73 (95% CI, 1.15 to 2.63, *p* = 0.009) in R‐B (interaction term *p* = 0.652). (Figure 1D,E). Patients with TMTV > 180 mL showed more frequent early progressions (POD24 58/310, 19%) compared to patients with lower TMTV (non POD24 42/354, 12%), *p* = 0.017.

In multivariable regression, either TMTV in continuous form or dichotomized at 180 mL, adjusted by FLIPI‐2, sex, treatment, and stratified by randomization arm, retained a prognostic role: HR 1.12 (95% CI 0.98 to 1.29, *p* = 0.089) by any increase of 500 mL for continuous TMTV and HR 1.38 (95% CI, 1.05 to 1.81, *p* = 0.020) for TMTV > 180 mL. Cox PH analysis is reported in Table 1.

In the multivariable Cox PH model of TMTV using the previously defined cut‐off of 510 mL, the HR was 1.14 (95% CI 0.83 to 1.57, *p* = 0.418) and 1.49 (95% CI 0.94 to 2.38, *p* = 0.092) in the reference arm (maintenance approach, see Tables S5 and S6).

### 3.3 Combining TMTV and FLIPI2

Multivariate analysis confirmed the prognostic role of the FLIPI‐2 score with a significantly worse PFS (HR 2.25, 95% CI 1.73 to 2.91, *p* < 0.001) for the experimental arm of the study. Moreover, the FLIPI2 score was superior to FLIPI in predicting both progression‐free survival (PFS) and overall survival (OS) (c‐Harrell 0.599 vs. 0.566 for PFS and 0.655 vs. 0.571 for OS).

**FIGURE 2:**
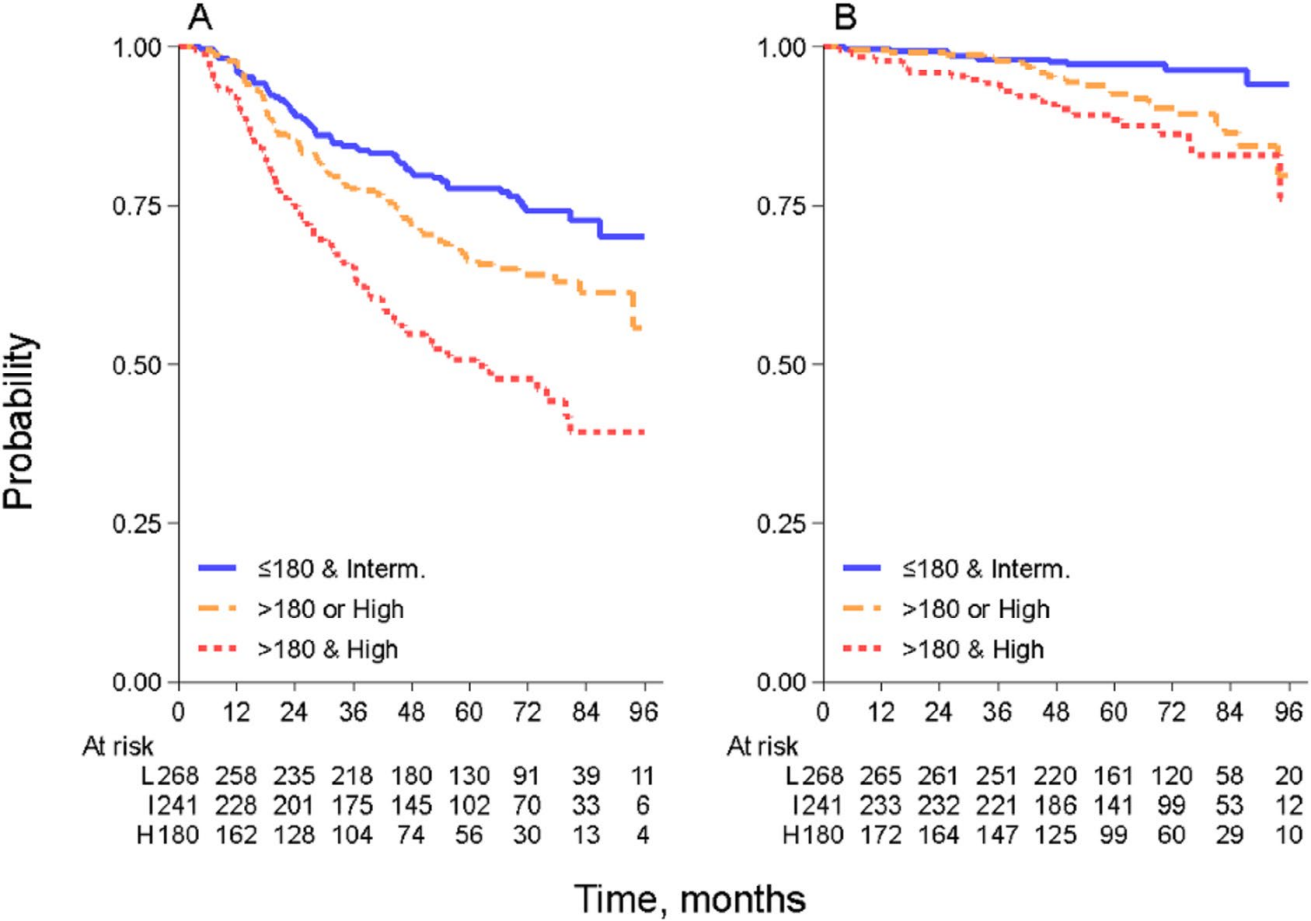
Progression‐free survival (A) and overall survival (B) stratified by groups obtained combining FLIPI‐2 and TMTV. [Color figure can be viewed at wileyonlinelibrary.com]

Combining TMTV and FLIPI2, patients were categorized into three distinct groups based on different PFS rates. The first group of patients with TMTV lower than 180 mL and intermediate FLIPI2 (*n* = 268, 39%) showed a 5‐year PFS of 78% (95% CI, 72 to 82%). The second group of patients with TMTV values higher than 180 mL or high‐risk FLIPI2 (*n* = 241,35%), had a 5‐year PFS rate of 66% (95% CI: 60–72) and an HR of 1.52 (95% CI: 1.09–2.11). Finally, patients with TMTV > 180 mL and high‐risk FLIPI2 (*n* = 180, 26%) had the worst PFS rate, with a 5‐year PFS rate of 51% (95% CI: 43–58), with an HR of 2.73 (95% CI: 1.98–3.78, *p* < 0.001). Comparing the high‐risk group to the intermediate‐risk group yielded an HR of 1.80 (95% CI: 1.33–2.44, *p* < 0.001). (Figure 2A). The addition of TMTV to FLIPI‐2 in the Cox PH model increased the c‐Harrell to 0.614 (95% CI, 0.577 to 0.650), compared with only FLIPI‐2 (0.597, 95% CI, 0.564 to 0.630) (Table 2). In the Supporting Information an internal validation with Out of the bag sample is reported (Table S4).

**TABLE 2:** Model with TMTV and FLIPI‐2 in progression‐free survival, stratified by randomization arm (*n* = 692).

Univariable	HR (95% CI)	*p*	c‐Harrell (95% CI)	
TMTV > 180	1.61 (1.24–2.09)	< 0.001	0.563 (0.530–0.596)	
FLIPI‐2 3/5	2.25 (1.73–2.91)	< 0.001	0.597 (0.564–0.630)	

Multivariable	HR (95% CI)	*p*	c‐Harrell (95% CI)	BIF (%)
TMTV> 180	1.37 (1.04–1.79)	0.024		62.6
FLIPI‐2 3/5	2.08 (1.59–2.72)	< 0.001		b 80.4
Model			0.614 (0.577–0.650)	
Difference	Model‐FLIPI‐2		0.016 (*p* = 0.046)	

*Note*: Cox PH regression stratified by randomization arm; c‐Harrell 95% CI estimated by means of jackknife method; BIF (Bootstrap inclusion fraction,1000 bootstrap resamples): number of times that TMTV> 180 mL was retained in model (with FLIPI‐2 fixed) with (a) likelihood ratio test < 0.05 and (b) AIC selection.

### 3.4 Overall Survival

In the study cohort, 57 deaths were reported (24 related to lymphoma), with 5‐year OS of 93% (95% CI, 91 to 95%). High FLIPI‐2 retained a strong prognostic value (HR 4.00, 95% CI 2.27 to 7.07, *p* < 0.001) while a trend towards a worst OS was observed for TMTV > 180 mL (HR 1.41, 95% CI 0.84 to 3.37, *p* = 0.197). The results of the combined model with TMTV and FLIPI2 are shown in Figure 2B. Patients with TMTV ≤ 180 mL and intermediate FLIPI2 had the highest 5‐year OS rate (97%;95% CI, 94 to 99%), followed by patients with TMTV greater than 180 mL or high‐risk FLIPI2 with a 5‐year OS rate of 93% (95% CI, 88 to 96%). Patients with TMTV > 180 mL and high‐risk FLIPI2 had the lowest 5‐year OS rate of 88% (95% CI 82 to 93%; < 0.001) as reported in Figure 2B.

## 4 Discussion

In this preplanned prospective evaluation of baseline PET scans of FL patients recruited in the randomized FOLL12 trial, we were able to demonstrate the strong and independent correlation between TMTV and the risk of progression or death. By categorizing patients into low and high TMTV groups using an optimal TMTV threshold of 180 mL, we found that those with high TMTV had significantly lower 5‐year PFS rates compared to the low TMTV group. The prognostic value of TMTV was independent of baseline characteristics and induction treatment, remaining significant in both the standard rituximab maintenance arm and the response‐adapted arm of the FOLL12 trial and in patients treated with R‐CHOP or R‐bandamustine. Additionally, integrating TMTV with the FLIPI2 score enhanced risk stratification, allowing us to identify three distinct risk groups. The group with high TMTV and high FLIPI2 scores exhibited the lowest progression‐free and overall survival rates, indicating a higher risk profile.

Despite its potential role as a prognostic tool, the application of TMTV in clinical trials has faced challenges mainly due to a lack of standardization in its definition [17]. The major ones included the use of a standardized procedure for patient scans and the use of harmonized PET scanners, both in terms of inter‐scanner calibration and image reconstruction, that are fulfilled by the FIL program for clinical trial qualification [21]. The method for lesion segmentation, which is a well‐known source of lesion volume variation [27], was addressed by the expert task force of the International Workshop on PET in Lymphoma and Myeloma of Menton, France. The experts' consensus agreed that a segmentation method using an SUV threshold of 4.0 yields consistent and reliable results in the FDG‐avid lymphoma setting, explicitly citing Hodgkin, B‐cell, and follicular lymphoma [17]. In this prospective study, we hence implemented the SUV4 cut‐off method for TMTV calculation. Our study is the first to apply the SUV4 cut‐off in a large cohort of treatment‐naïve FL patients.

Our findings align with previously published retrospective studies by Meignan et al. and Cotterau et al. [13], which both employed the SUV 41% method for TMTV calculation and utilized a cut‐off value of 510 mL, as initially reported by Meignan [9]. Notably, our study has established a lower TMTV cutoff value of 180 mL compared to the previously published value of 510 mL by Meignan et al. [11]. A major reason for this is the difference threshold used for segmentation. In Cotterau's study, median baseline TMTV was 284 mL (Q1‐Q3 129–526 mL and 148–562 mL for standard and experimental arm, aggregated data are not available) and median SUVmax 11.28. In Meignan's study, median baseline TMTV was 297 mL (Q1‐Q3 131–567 mL) and median SUVmax 10. In our study, median TMTV was 162 mL (Q1‐Q3 50–388) and median SUVmax 9.8 (Q1‐Q3 7.4–13.4). It is likely that the threshold is lower in our case. Considering that, as agreed in the Menton meeting and as stated in Barrington et al. [28] and Boellaard et al. [17] we believe that our threshold could be used in next studies as a reference and could be tested in a similar patient population. However, it is important to acknowledge that TMTV range in the current dataset differs from those in the Meignan study, which may influence the derivation of a “single cutoff.” This is a well‐known limitation, as using a single cutoff for continuous variables can inherently lead to different results depending on the range and distribution of the data [29].

Our results, along with the two other aforementioned positive studies, are in contrast with the TMTV analysis conducted in patients enrolled in the GALLIUM, which used a SUV_max_ ≥ 2.5 and SUV_max_ ≥ 41% threshold to calculate TMTV [12]. Unfortunately, the primary analysis of the study was not able to confirm the prognostic role of TMTV, but a full report has never been published to obtain enough information to depict a reason for this discrepancy. Interestingly, a subsequent analysis of the same population by the same research team was able to find a prognostic value of baseline TMTV for PFS when a different and fully automated segmentation algorithm was used [30]. It is worth noting that we employed a semi‐automatic approach to segment the lesions, wherein the expert reviewers manually excluded regions unrelated to lymphoma but did not add or modify any regions of interest to ensure consistency in TMTV calculation. While this approach is not entirely automated, it is quite close to being so, and we can assume that the level of automation achieved is very high.

An important finding of our study is the confirmation of the prognostic value of the FLIPI‐2 score. In our cohort FLIPI2 score outperformed FLIPI in terms of predicting both PFS and OS. These results are in line with the results reported by Meignan et al. Furthermore, our results suggest that the combination of TMTV and FLIPI‐2 score could improve risk stratification and make treatment decisions more accurate for FL patients. We found that patients with TMTV higher than 180 mL and high‐risk FLIPI‐2 had the worst PFS rate, with a 5‐year PFS of 50% and a hazard ratio of 2.93. Importantly, the addition of TMTV to FLIPI‐2 in the Cox proportional hazards model improved the c‐Harrell statistic, improving the accuracy in PFS prediction.

Our cohort demonstrated a 5‐year OS rate of 92% (95% CI, 89 to 94%), which is consistent with previous research. However, 58 deaths were observed in our cohort, indicating the importance of identifying and managing high‐risk patients. Although TMTV alone showed a weak prognostic role for OS, combining TMTV and FLIPI‐2 improved survival prediction accuracy.

Our study has several strengths. Firstly, it is the largest prospective study to date that has investigated the prognostic value of TMTV in a cohort of FL patients. Secondly, the study population was taken from a randomized controlled trial with long‐term follow‐up in which the collection of baseline PET was planned and standardized by the protocol. Thirdly, we used a standardized and validated method to measure TMTV, ensuring reproducibility of results. Conversely, our study has some limitations that should be considered when interpreting our findings. Firstly, we used a semiautomatic approach for TMTV computation; it should be highlighted that a fully automated technique for TMTV assessment could potentially enhance reproducibility and facilitate prompt reporting of results following a PET/CT scan. Secondly, the FOLL12 trial included only patients with high tumor burden advanced‐stage FL. Therefore, our results may not be applicable to patients with low tumor burden FL. Lastly, we lack an external validation of our TMTV cut‐off point. However, to ensure the accuracy of our findings, similarly to what was initially done by Meignan et al. [9] we employed a bootstrap resampling method, computing scores in the bootstrap sample and then validating the same scores out‐of‐bootstrap sample, avoiding potential biases in the results.

In conclusion, our findings indicate that baseline TMTV defined using the new SUV4 standard for contouring lymphoma sites is a robust predictor of survival outcomes in FL. Our study suggests that TMTV and FLIPI‐2 can be used in combination to improve risk stratification and treatment decisions for FL patients, particularly for patients who are at high risk of disease progression and may require more aggressive treatment strategies.

## Author Contributions

Conception and design: S.L., A.V., L.G., S.C., M.F. Provision of study materials or patients: S.L., S.P., A.A., C.B., P.C., J.O., L.A., F.A., A.I., L.N., V.T., A.P., C.S., A.P., F.C., S.M., E.C., N.D.R., C.M., A.P., P.Z., C.B., F.R., A.M.C. Collection and assembly of data: R.D., F.B., F.F. Data analysis and interpretation: R.D., S.L., A.V., L.M., L.G., S.C., F.F. Manuscript writing: R.D., S.L., L.G., A.V., C.M. Final approval of manuscript: All authors.

## Conflicts of Interest

The authors declare no conflicts of interest.

## Supporting information


**Table S1.** Distribution of patient’s characteristics between cases included and excluded in TMTV analysis.
**Table S2.** TMTV distribution by patient’s and study characteristics.
**Figure S1.** Maximum logrank statistic of TMTV adjusted by FLIPI‐2, sex, induction treatment and stratified by randomization arm.
**Figure S2.** Choice of the number of knots.
**Figure S3.** Cox PH model with TMTV4 modeled with RCS (5 knots), adjusted by FLIPI‐2, sex, induction treatment and stratified by randomization arm.
**Table S3.** A Interaction of 180 mL cut‐off obtained by AUC‐ROC and RCS Cox PH model with the 240 mL cut‐off of longrank test method.
**Figure S4.** PFS of study arm by the 180 mL and 240 mL TMTV cutoff.
**Table S4.** Out of bootstrap (OOB) sample from original cohort of 689 case, TMTV4 > 180 mL.
**Figure S5.** PFS by our study TMTV cutoff (180 mL) and previous published studies (510 mL).
**Table S5.** Comparison of our study 180 mL TMTV cutoff with the previous published 510 mL cutpoint.
**Table S6.** Classification table at 3‐yrs PFS in reference arm (rituximab maintenance).

## Data Availability

Individual participant data that underlie the results reported in this article, after de‐identification, can be made available to investigators for research purposes on a case‐by‐case basis following the time of this publication and in accordance with the FIL Data Sharing Policy.

## References

[1] L. M. Morton , S. S. Wang , S. S. Devesa , P. Hartge , D. D. Weisenburger , and M. S. Linet , “Lymphoma Incidence Patterns by WHO Subtype in the United States, 1992‐2001,” Blood 107, no. 1 (2006): 265–276, 10.1182/BLOOD-2005-06-2508. 16150940 PMC1895348

[2] F. Cabanillas , “Curability of Advanced Indolent or Low‐Grade Follicular Lymphomas: Time for a New Paradigm?,” Journal of Clinical Oncology 31 (2013): 14–16. 23045578 10.1200/JCO.2012.41.7527

[3] C. Casulo , “Prognostic Factors in Follicular Lymphoma: New Tools to Personalize Risk,” Hematology: The American Society of Hematology Education Program 2016, no. 1 (2016): 269, 10.1182/ASHEDUCATION-2016.1.269. PMC6142481 27913491

[4] P. Solal‐Celigny , P. Roy , P. Colombat , et al., “Follicular Lymphoma International Prognostic Index,” Blood 104, no. 5 (2004): 1258–1265, 10.1182/blood-2003-12-4434. 15126323

[5] M. Federico , M. Bellei , L. Marcheselli , et al., “Follicular Lymphoma International Prognostic Index 2: A New Prognostic Index for Follicular Lymphoma Developed by the International Follicular Lymphoma Prognostic Factor Project,” Journal of Clinical Oncology 27 (2009): 4555–4562. 19652063 10.1200/JCO.2008.21.3991

[6] L. Guerra , S. Chauvie , F. Fallanca , et al., “End of Induction [18F]FDG PET Is Prognostic for Progression‐Free Survival and Overall Survival in Follicular Lymphoma Patients Enrolled in the FOLL12 Trial,” European Journal of Nuclear Medicine and Molecular Imaging 51, no. 11 (2024): 3311–3321, 10.1007/S00259-024-06765-Z. 38795120

[7] S. Luminari , A. Versari , A. Franceschetto , et al., “The Role of Qualitative FDG‐PET Assessment Using the 5 Point Deauville Scale for Postinduction Response Assessment in Patients With Follicular Lymphoma. A Study From the Fondazione Italiana Linfomi (FIL),” Blood 122 (2013): 4295.

[8] J. Dupuis , A. Berriolo‐Riedinger , A. Julian , et al., “Impact of [18F]Fluorodeoxyglucose Positron Emission Tomography Response Evaluation in Patients With High‐Tumor Burden Follicular Lymphoma Treated With Immunochemotherapy: A Prospective Study From the Groupe D'etudes Des Lymphomes de L'adulte and GOELAMS,” Journal of Clinical Oncology 30, no. 35 (2012): 4317–4322, 10.1200/JCO.2012.43.0934. 23109699

[9] J. Trotman , S. Luminari , S. Boussetta , et al., “Prognostic Value of PET‐CT After Fi Rst‐Line Therapy in Patients With Follicular Lymphoma: A Pooled Analysis of Central Scan Review in Three Multicentre Studies,” Lancet Haematology 1, no. 1 (2014): e17–e27, 10.1016/S2352-3026(14)70008-0. 27030064

[10] L. Kostakoglu and S. Chauvie , “Metabolic Tumor Volume Metrics in Lymphoma,” Seminars in Nuclear Medicine 48, no. 1 (2018): 50–66, 10.1053/J.SEMNUCLMED.2017.09.005. 29195618

[11] M. Meignan , A. S. Cottereau , A. Versari , et al., “Baseline Metabolic Tumor Volume Predicts Outcome in High‐Tumor‐Burden Follicular Lymphoma: A Pooled Analysis of Three Multicenter Studies,” Journal of Clinical Oncology 34, no. 30 (2016): 3618–3626, 10.1200/JCO.2016.66.9440. 27551111

[12] S. F. Barrington , J. Trotman , D. Sahin , et al., “Baseline PET‐Derived Metabolic Tumor Volume Metrics Did Not Predict Outcomes in Follicular Lymphoma Patients Treated With First‐Line Immunochemotherapy and Antibody Maintenance in the Phase III GALLIUM Study,” Blood 132, no. Supplement 1 (2018): 2882, 10.1182/BLOOD-2018-99-117235.

[13] A. S. Cottereau , L. Rebaud , J. Trotman , et al., “Metabolic Tumor Volume Predicts Outcome in Patients With Advanced Stage Follicular Lymphoma From the RELEVANCE Trial,” Annals of Oncology 35, no. 1 (2024): 130–137, 10.1016/J.ANNONC.2023.10.121. 37898239

[14] S. Luminari , “Personalised Approach in Follicular Lymphoma,” Lancet Oncology 19, no. 11 (2018): 1431–1432, 10.1016/S1470-2045(18)30688-0. 30309757

[15] J. H. Liang , Y. P. Zhang , J. Xia , et al., “Prognostic Value of Baseline and Interim Total Metabolic Tumor Volume and Total Lesion Glycolysis Measured on 18F‐FDG PET‐CT in Patients With Follicular Lymphoma,” Cancer Research and Treatment: Official Journal of Korean Cancer Association 51, no. 4 (2019): 1479–1487, 10.4143/CRT.2018.649. PMC6790864 30913868

[16] S. Luminari , M. Manni , S. Galimberti , et al., “Response‐Adapted Postinduction Strategy in Patients With Advanced‐Stage Follicular Lymphoma: The FOLL12 Study,” Journal of Clinical Oncology 40, no. 7 (2022): 729–739, 10.1200/JCO.21.01234. 34709880

[17] R. Boellaard , I. Buvat , C. Nioche , et al., “International Benchmark for Total Metabolic Tumor Volume Measurement in Baseline 18F‐FDG PET/CT of Lymphoma Patients: A Milestone Toward Clinical Implementation,” Journal of Nuclear Medicine 65, no. 9 (2024): 1343–1348, 10.2967/JNUMED.124.267789. 39089812 PMC11372260

[18] P. Brice , Y. Bastion , E. Lepage , et al., “Comparison in Low‐Tumor‐Burden Follicular Lymphomas Between an Initial No‐Treatment Policy, Prednimustine, or Interferon Alfa: A Randomized Study From the Groupe D'etude Des Lymphomes Folliculaires. Groupe D'etude Des Lymphomes de L'adulte,” Journal of Clinical Oncology 15, no. 3 (1997): 1110–1117, 10.1200/JCO.1997.15.3.1110. 9060552

[19] B. D. Cheson , R. I. Fisher , S. F. Barrington , et al., “Recommendations for Initial Evaluation, Staging, and Response Assessment of Hodgkin and Non‐Hodgkin Lymphoma: The Lugano Classification,” Journal of Clinical Oncology 32, no. 27 (2014): 3059–3067, 10.1200/JCO.2013.54.8800. 25113753 PMC4979083

[20] R. Boellaard , R. Delgado‐Bolton , W. J. G. Oyen , et al., “FDG PET/CT: EANM Procedure Guidelines for Tumour Imaging: Version 2.0,” European Journal of Nuclear Medicine and Molecular Imaging 42, no. 2 (2015): 328–354, 10.1007/s00259-014-2961-x. 25452219 PMC4315529

[21] S. Chauvie , F. Bergesio , F. Fioroni , et al., “The (68)Ge Phantom‐Based FDG‐PET Site Qualification Program for Clinical Trials Adopted by FIL (Italian Foundation on Lymphoma),” Physica Medica 32, no. 5 (2016): 651–656, 10.1016/J.EJMP.2016.04.004. 27133138

[22] S. Chauvie , A. Biggi , A. Stancu , et al., “WIDEN: A Tool for Medical Image Management in Multicenter Clinical Trials,” Clinical Trials 11, no. 3 (2014): 355–361, 10.1177/1740774514525690. 24711610

[23] M. Meignan , M. Sasanelli , R. O. Casasnovas , et al., “Metabolic Tumour Volumes Measured at Staging in Lymphoma: Methodological Evaluation on Phantom Experiments and Patients,” European Journal of Nuclear Medicine and Molecular Imaging 41, no. 6 (2014): 1113–1122, 10.1007/s00259-014-2705-y. 24570094

[24] B. Lausen and M. Schumacher , “Maximally Selected Rank Statistics,” Biometrics 48, no. 1 (1992): 73, 10.2307/2532740.

[25] P. J. Heagerty , T. Lumley , and M. S. Pepe , “Time‐Dependent ROC Curves for Censored Survival Data and a Diagnostic Marker,” Biometrics 56, no. 2 (2000): 337–344, 10.1111/j.0006-341X.2000.00337.x. 10877287

[26] F. E. Harrell , Regression Modeling Strategies (Springer, 2015), 10.1007/978-3-319-19425-7.

[27] S. Kanoun , I. Tal , A. Berriolo‐Riedinger , et al., “Influence of Software Tool and Methodological Aspects of Total Metabolic Tumor Volume Calculation on Baseline [18F]FDG PET to Predict Survival in Hodgkin Lymphoma,” PLoS One 10, no. 10 (2015): e0140830, 10.1371/journal.pone.0140830. 26473950 PMC4608733

[28] S. F. Barrington and M. Meignan , “Time to Prepare for Risk Adaptation in Lymphoma by Standardizing Measurement of Metabolic Tumor Burden,” Journal of Nuclear Medicine 60, no. 8 (2019): 1096–1102, 10.2967/JNUMED.119.227249. 30954945 PMC6681699

[29] N. G. Mikhaeel , M. W. Heymans , J. J. Eertink , et al., “Proposed New Dynamic Prognostic Index for Diffuse Large B‐Cell Lymphoma: International Metabolic Prognostic Index,” Journal of Clinical Oncology 40, no. 21 (2022): 2352–2360. 35357901 10.1200/JCO.21.02063 PMC9287279

[30] S. Jemaa , J. Fredrickson , A. Coimbra , et al., “A Fully Automated Measurement of Total Metabolic Tumor Burden in Diffuse Large B‐Cell Lymphoma and Follicular Lymphoma,” Blood 134 (2019): 4666, 10.1182/blood-2019-124793.

