# Supplementary material for: Total Metabolic Tumor Volume Is a Strong Independent Prognostic Factor in Follicular Lymphomas: Results From a Sub‐Study of the FOLL12 Trial
Source: Am J Hematol. 2025 May 14;100(7):1196–204. doi: 10.1002/ajh.27711 (PMC12146809; doi:10.1002/ajh.27711)
Supplement: Supplementary file 1 — Table S1. Distribution of patient’s characteristics between cases included and excluded in TMTV analysis. Table S2. TMTV distribution by patient’s and study characteristics. Figure S1. Maximum logrank statistic of TMTV adjusted by FLIPI‐2, sex, induction treatment and stratified by randomization arm. Figure S2. Choice of the number of knots. Figure S3. Cox PH model with TMTV4 modeled with RCS (5 knots), adjusted by FLIPI‐2, sex, induction treatment and stratified by randomization arm. Table S3. A Interaction of 180 mL cut‐off obtained by AUC‐ROC and RCS Cox PH model with the 240 mL cut‐off of longrank test method. Figure S4. PFS of study arm by the 180 mL and 240 mL TMTV cutoff. Table S4. Out of bootstrap (OOB) sample from original cohort of 689 case, TMTV4 > 180 mL. Figure S5. PFS by our study TMTV cutoff (180 mL) and previous published studies (510 mL). Table S5. Comparison of our study 180 mL TMTV cutoff with the previous published 510 mL cutpoint. Table S6. Classification table at 3‐yrs PFS in reference arm (rituximab maintenance). [file AJH-100-1196-s001.docx]

**- Appendix**

**Table S1:** Distribution of patient’s characteristics between cases included and excluded in TMTV analysis

| **Covariate** | **Exluded (N=94)** | **Included (N=692)** | **p** |
| --- | --- | --- | --- |
|  | **n (%)** | **n (%)** |  |
| Age >60 | 47 (50) | 344 (50) | 1.000 |
| Sex male | 53 (56) | 319 (46) | 0.062 |
| B2M>ULN | 48 (51) | 379 (55) | 0.510 |
| BM + | 56 (60) | 381 (55) | 0.440 |
| LoDLIN >6 cm | 51 (54) | 384 (55) | 0.826 |
| Hb <12 mg/dL | 15 (16) | 112 (16) | 1.000 |
| FLIPI-2 High | 38 (40) | 278 (40) | 1.000 |
| Experimental arm | 36 (38) | 357 (52) | 0.021 |
| R-B | 49 (52) | 292 (42) | 0.076 |

**Table S2.** TMTV distribution by patient’s and study characteristics

|  |  |  | **TMTV value** |  |
| --- | --- | --- | --- | --- |
| **Factor** |  | **n (%)** | **Median (IQR)** | **P-value** |
| Overall |  | 689 | 161 (51-387) | - |
| Age, year | $\leq$60 | 348 (50) | 158 (59-370) | 0.807 |
|  | >60 | 341 (50) | 161 (44-409) |  |
| Sex | M | 317 (46) | 142 (41-409) | 0.154 |
|  | F | 372 (54) | 176 (65-374) |  |
| Bone marrow | - | 308 (45) | 130 (40-314) | <0.001 |
|  | + | 381 (55) | 200 (62-481) |  |
| B2M | >ULN | 311 (45) | 108 (35-232) | <0.001 |
|  | $\leq$ULN | 378 (55) | 239 (77-523) |  |
| LoDLIN | $\leq$6 cm | 307 (45) | 81 (21-273) | <0.001 |
|  | >6 cm | 382 (55) | 219 (97-476) |  |
| Hemoglobin | ≥12 mg/dL | 578 (84) | 149 (42-354) | <0.001 |
|  | <12 mg/dL | 111 (16) | 288 (108-615) |  |
| Stage | II | 77 (11) | 85 (28-202) | <0.001 |
|  | III | 141 (21) | 145 (51-340) |  |
|  | IV | 469 (68) | 181 (61-433) |  |
| FLIPI-2 | 1/2 | 411 (60) | 104 (33-258) | <0.001 |
|  | 3/5 | 278 (40) | 283 (113-600) |  |
| FLIPI | 0/1 | 161 (24) | 90 (34-221) | <0.001 |
|  | 2 | 279 (42) | 139 (44-361) |  |
|  | 3/5 | 225 (34) | 266 (101-578) |  |
| Bcl2 | No | 304 (44) | 167 (44-380) | 0.679 |
|  | Yes | 380 (56) | 154 (52-388) |  |
| Arm | Reference | 334 (48) | 171 (58-397) | 0.349 |
|  | Experimental | 355 (52) | 147 (47-376) |  |
| Treatment | R-CHOP | 399 (58) | 166 (51-421) | 0.312 |
|  | R-B | 290 (42) | 153 (48-363) |  |

B2M: beta2 microglobuline; LoDLIN: longest diameter of the largest involved node; FLIPI: follicular lymphoma international prognostic index; ULN: upper limit of normality; R-CHOP: R-CHOP, rituximab plus cyclophosphamide, doxorubicin, vincristine, and prednisone; RB: rituximab plus bendamustine

**1- Selection of cut-off**

Three different approaches were used to define the cutoff for progression free survival (PFS) prediction for total metabolic tumor volume (TMTV), startified by random arm:

a) Maximum log-rank statistic;

b) Area under curve-receiver operating characteristic (AUC-ROC) analysis for survival data;

c) Cox proportional hazard model with TMTV4 modelled with restricted cubic spline (RCS) used to evaluate the continuous nonlinear relationship between TMTV and PFS risk. The knots for the restricted cubic spline function were selected by minimun AIC (Akaike index criterion) after 1000 bootstrap resample, with knots ranging from 3 to 7 and placed at quantiles position as recommend by F. Harrell.

The cut-off was internally vaidated by means of bootstrap procedure: from the bootstrap samples we evaluated the repeatability of cut-off selected by RCS, while in the out of bootstrap sample (OOB, test set that exclude about 37% of record from original data) evaluated the possible reproducibility of the cut-off expressed as HR with 95%CI.

**a - Maximum logrank statistic**, TMTV adjusted by FLIPI-2, sex, induction treatment and stratified by randomization arm: Optimal cut-point TMT4 = 234 mL (Figure S1)

*B Lausen, M Schumacher: Maximally Selected Rank Statistics. Biometrics, 1992, 48, 73–85*

**Figure S1**: Maximum logrank statistic of TMTV adjusted by FLIPI-2, sex, induction treatment and stratified by randomization arm

**b- AUC-ROC**

**- at 3-years PFS**

Optimal cutpoint with Lui criterion TMTV4 = 171 mL

Sensitivity 57%, Specificity 55%, AUC-ROC 0.58

**- at 4-years PFS**

Optimal cutpoint with Lui criterion TMTV4 = 181 mL

Sensitivity 57%, Specificity 57%, AUC-ROC 0.58

*PJ Heagerty, T Lumley, MS Pepe: Time-dependent ROC curves for censored survival data and a diagnostic marker. Biometrics, 2000, 56: 337-344*] according with Liu method [*WJ Youden Index for rating diagnostic tests. 1950, Cancer 3: 32-35*

**c- Cox model with TMTV4 modelled with RCS**, adjusted by FLIPI-2, sex, induction treatment and stratified by randomization arm

**Figure S2**: Choice of the number of knots

#5: knots at “5 27.5 50 72.5 95” percentile of distribution

**Figure S3:** Cox PH model with TMTV4 modelled with RCS (5 knots), adjusted by FLIPI-2, sex, induction treatment and stratified by randomization arm:

The HR as function of TMTV4 change quickly around 180 mL (HR crosses the value of one with TMTV4 = 181 mL).

*F. Harrell: regression modeling strategies; 2nd edition, Springer, 2015*

**2- Choice of the cut-off**

AUC-ROC and RCS Cox PH model gave two similar cut-offs (171 at 3-years and 181 at 4-years for AUC-ROC and and 180 ml by Cox model) and we selected 180 mL.

Instead, maximum logrank test selected 235 mL, that we rounded as 240 mL.

**Table S3**: Interaction of 180ml cut-off obtained by AUC-ROC and RCS Cox PH model with the 240mL cut-off of longrank test method

| **Cut-off** | **≤240 mL** | **>240 mL** | **Total** |
| --- | --- | --- | --- |
| **≤180 mL** | 366 (53%) | 0 | 366 (53%) |
| **> 180 mL** | 61 (9%) | 262 (38%) | 323 (47%) |
| **Total** | 427 (61%) | 262 (38%) | 689 |

Percentage by cells

**Figure S4:** PFS of study arm by the 180mL and 240mL TMTV cutoff

Patients with baseline TMTV >180 and <240 mL were split in the worse prognostic group in the reference arm, while they were merged in the better prognostic group in the experimental arm.

Since the reference arm, which included rituximab maintenance that is currently acknowledged as the standard choice for high tumor burden FL patients, we selected the value of 180 mL as the best cut-off for the patient population.

**3) Stability and repeatability of cut-point 180 mL**

After 1000 bootstrap resamples, the median cut-off was of 175 mL with a bias of 2.8% on reference of 180 mL.

**Table S4**: out of bootstrap (OOB) sample from original cohort of 689 case, TMTV4 >180 mL:

| **OOB sample** | **mean** |  |
| --- | --- | --- |
| **Sample size** | 253 |  |
| **Failure,** | 85 |  |
| **OOB HR (95%CI)** | 1.68 (1.09-2.60) | p = 0.028 |
| **Bootstrap sample** | **HR (95%CI)** |  |
| **Bootstrap sample A** | 1.66 (1.28-2.15) | p <0.001 |
| **Bootstrap sample B** | 1.40 (1.06-1.84) | p = 0.020 |

A: TMTV>180 mL stratified by randomization arm; B: TMTV>180 adjusted by FLIPI-2, sex, induction treatment and stratified by randomization arm

The cut-point showed a acceptable stability and repeatability from internal validation.

**4- Comparison with cut-off of 510 mL**

**Figure S5**: PFS by our study TMTV cutoff (180mL) and previous published studies (510 mL).

**Table S5:** Comparison of our study 180 mL TMTV cutoff with the previous published 510mL cutpoint

|  |  | **Cut-point** | |
| --- | --- | --- | --- |
| **Parameter** |  | **>180 mL** | **>510 mL** |
| Worse group, n (%) |  | 323 (47%) | 127 (18%) |
| All HR (95%CI), p | Only stratified by arm | 1.65 (1.27-2.14), p<0.001 | 1.50 (1.11-2.03), p=0.009 |
| c-Harrel % (95%CI) | Only stratified by arm | 56.3 (53.0-59.6) | 53.4 (50.6-56.1) |
|  |  |  |  |
| **REF** HR (95%CI), p |  | 1.84 (1.21-2.81), p=0.005 | 2.05 (1.31-3.23), p=0.002 |
| Test HR 510 vs 180 | In reference arm | 1.12 (0.72-1.73), p=0.621 | |
| **EXP** HR (95%CI), p |  | 1.55 (1.11-2.15), p=0.010 | 1.19 (0.78-1.81), p=0.412 |
|  |  |  |  |
| HR (95%CI) | Multiple Cox regression | 1.38 (1.05-1.81), p=0.020 | 1.14 (0.83-1.57), p=0.418 |
| c-Harrell % (95%CI) | Multiple Cox regression | 63.0 (58.9-66.4) | 62.0 (58.2-65.8) |

Multiple Cox regression: TMTV4 adjusted by FLIPI-2, sex, induction treatment and stratified by randomization arm.

**Table S6**: Classification table at 3-yrs PFS in reference arm (rituximab maintenance)

| **Parameter** | **>180 mL** | **>510 mL** |
| --- | --- | --- |
| Sensitivity (true positive) | 61.8% | 27.3% |
| Specificity (true negative) | 53.8% | 82.9% |
| Positive predictive value | 21.8% | 25.0% |
| Negative predictive value | 87.1% | 84.6% |
| False positive rate | 46.2% | 17.0% |
| False negative rate | 38.2% | 72.7% |
| AUC-ROC | 57.8% | 55.1% |

The cut-off of 510 mL retained a good prognostic role in reference arm (with maintenance), but 180 mL showed a higher flexibility, collected more patients in the worse prognostic group and showed a comparable HR with 510 mL. Also, the cut-off of 180 mL showed a better sensitivity and tended to give an alert about patients who were at higher risk of progression or death for any cause, which was the primary endpoint of our study.
